# MAGI1 inhibits interferon signaling to promote influenza A infection

**DOI:** 10.3389/fcvm.2022.791143

**Published:** 2022-08-23

**Authors:** Yin Wang, Jun-ichi Abe, Khanh M. Chau, Yongxing Wang, Hang Thi Vu, Loka Reddy Velatooru, Fahad Gulraiz, Masaki Imanishi, Venkata S. K. Samanthapudi, Minh T. H. Nguyen, Kyung Ae Ko, Ling-Ling Lee, Tamlyn N. Thomas, Elizabeth A. Olmsted-Davis, Sivareddy Kotla, Keigi Fujiwara, John P. Cooke, Di Zhao, Scott E. Evans, Nhat-Tu Le

**Affiliations:** ^1^Department of Cardiology, The University of Texas MD Anderson Cancer Center, Houston, TX, United States; ^2^Department of Cardiovascular Sciences, Center for Cardiovascular Regeneration, Houston Methodist Research Institute, Houston, TX, United States; ^3^Department of Pulmonary Medicine, The University of Texas MD Anderson Cancer Center, Houston, TX, United States

**Keywords:** IAV, MAGI1, MX1, interferon signaling, IRF3, EC inflammation

## Abstract

We have shown that membrane-associated guanylate kinase with inverted domain structure-1 (MAGI1), a scaffold protein with six PSD95/DiscLarge/ZO-1 (PDZ) domains, is involved in the regulation of endothelial cell (EC) activation and atherogenesis in mice. In addition to causing acute respiratory disease, influenza A virus (IAV) infection plays an important role in atherogenesis and triggers acute coronary syndromes and fatal myocardial infarction. Therefore, the aim of this study is to investigate the function and regulation of MAGI1 in IAV-induced EC activation. Whereas, EC infection by IAV increases MAGI1 expression, MAGI1 depletion suppresses IAV infection, suggesting that the induction of MAGI1 may promote IAV infection. Treatment of ECs with oxidized low-density lipoprotein (OxLDL) increases MAGI1 expression and IAV infection, suggesting that MAGI1 is part of the mechanistic link between serum lipid levels and patient prognosis following IAV infection. Our microarray studies suggest that MAGI1-depleted ECs increase protein expression and signaling networks involve in interferon (IFN) production. Specifically, infection of MAGI1-null ECs with IAV upregulates expression of signal transducer and activator of transcription 1 (STAT1), interferon b1 (IFNb1), myxovirus resistance protein 1 (MX1) and 2′-5′-oligoadenylate synthetase 2 (OAS2), and activate STAT5. By contrast, MAGI1 overexpression inhibits *Ifnb1* mRNA and MX1 expression, again supporting the pro-viral response mediated by MAGI1. MAGI1 depletion induces the expression of MX1 and virus suppression. The data suggests that IAV suppression by MAGI1 depletion may, in part, be due to MX1 induction. Lastly, interferon regulatory factor 3 (IRF3) translocates to the nucleus in the absence of IRF3 phosphorylation, and IRF3 SUMOylation is abolished in MAGI1-depleted ECs. The data suggests that MAGI1 inhibits IRF3 activation by maintaining IRF3 SUMOylation. In summary, IAV infection occurs in ECs in a MAGI1 expression-dependent manner by inhibiting anti-viral responses including STATs and IRF3 activation and subsequent MX1 induction, and MAGI1 plays a role in EC activation, and in upregulating a pro-viral response. Therefore, the inhibition of MAGI1 is a potential therapeutic target for IAV-induced cardiovascular disease.

## Highlights

- IAV infection increases MAGI1 expression.
- MAGI1 depletion upregulates anti-viral response including STATs and IRF3 activation and inhibits EC infection by IAV.
- OxLDL induces MAGI1 expression and amplifies IAV infection in ECs.
- Anti-viral effects of MAGI1 depletion is at least partially depends on MX1 induction.
- De-SUMOylation and the consequent activation of IRF3 are induced by MAGI1 depletion.
- MAGI1 plays a crucial role in both accelerating EC activation and IAV infection.

## Introduction

Whereas, three types of influenza viruses (A, B, and C) (1), negative-strand RNA viruses belonging to the *Orthomyxoviridae* family, infect humans, seasonal flu epidemics are usually caused by influenza A virus (IAV) (2, 3), which targets epithelial cells in the human respiratory tract. The gap between the airspace and the capillary ECs is ~0.5 μm, and alveolar wall cells and capillary ECs are separated only by the basal lamina and so that the endothelium is likely to be also exposed to free virus particles during IAV infection (4). According to some studies, IAV infects ECs *in vivo*, in association with development of flu symptom (5–8). However, other studies have suggested that ECs indirectly contribute to the pathogenesis of virus infection through control of the local inflammatory milieu in the lungs (9, 10). Indeed, some studies implicate EC function in IAV pathogenesis; i.e., in addition to causing acute respiratory disease IAV may trigger acute coronary syndromes and fatal myocardial infarction (11–15). Also, IAV infection of the vascular wall and/or increased levels of circulating pro-inflammatory cytokines may contribute to atherosclerotic lesion progression (16, 17). However, the molecular pathway by which IAV promotes atherogenesis has not been identified so that these processes may simply share common risk factors (10, 17).

The severity of IAV infection depends on the host inflammatory response. During inflammation, EC activation is a key step. Our previous studies showed that MAGI1 depletion prevents EC activation induced by pro-atherogenic type of flow (18–20). MAGI1, a scaffold protein with six PDZ domains, one guanylate kinase domain, and two WW (rsp5) domains flanked by the first and second PDZ domains, localizes to the tight and adherens junctions (20–22). A genome-wide association study revealed a strong association between MAGI1 locus and various chronic inflammatory diseases including Crohn's disease, in which MAGI1-dependent maintenance of the tight seal of the gastrointestinal tract is compromised (23). Indeed, we also showed that MAGI1 plays a critical role in regulating EC permeability (19).

Although the role of MAGI1 in EC activation has been reported (18–20), its role in IAV-induced EC activation is unknown. The aim of the study was to test the hypothesis that MAGI1 drives EC activation after IAV infection, and that this process promotes IAV-mediated cardiovascular disease.

## Experimental procedures

### Mice

All mice were fed a normal diet and housed in a room with an ambient temperature of 22°C and a 12-h light/12-h dark cycle in a pathogen-free environment at the Texas A&M Institute of Biosciences and Technology. MAGI1^−/−^ (homozygous knocked out) mice were generated and characterized as we have previously described (20). C57BL/6 mice were purchased from the Jackson Laboratory (Bar Harbor, ME). First, MAGI1^−/−^ mice were crossed with C57BL/6 mice to generate MAGI1^−/+^ (heterozygous) mice, which were then used to generate MAGI1^−/−^ and the wild type (WT) littermate control mice. All procedures on mice were approved by the Institutional Care and Use Committees of the Texas A&M Institute of Biosciences and Technology (2014-0231, 2017-0154) and The University of Texas MD Anderson Cancer Center (00001652, 00001109).

### Cells

HUVECs were isolated through a collagenase digestion of the endothelium of human umbilical cord veins and were grown on culture dishes coated with 0.2% gelatin type A (MP Biomedicals, Solon, OH, USA) in Endothelial Cell Medium (ECM, Catalog #1001, Science Cell, San Diego, CA, USA). HUVEC isolation was approved by the Houston Methodist Research Institute (HMRI) Institutional Review Board (IRB, permit No. Pro00020559). Mouse lung ECs were isolated as we have performed and described previously (17, 22, 23). Mouse lung EC isolation was approved by the HMRI Institutional Animal Care and Use Committee (IACUC, permit No. IS00006725). Briefly, lungs were harvested from 6 to 8 weeks old mice, washed thoroughly in cold PBS, minced finely with scissors, then digested by collagenase. Sheep anti-rat PECAM-1-conjugated Dynabeads (Catalog #11035, Invitrogen, Carlsbad, CA, USA) were prepared and used to isolate mouse lung ECs. Mouse lung ECs were cultured in DMEM (Catalog #SH30243.0, Hyclone, Logan, UT, USA) supplemented with 20% FBS (Catalog #F2442, Sigma-Aldrich, Saint Louis, MO), 1% EC growth supplement (Promo Cell, Heidelberg, Germany), 25 mM HEPES buffer (Catalog #25-060-CI, Corning, Manassas, VA, USA), 1% non-essential amino acid solution (Catalog #25-025-CI, Corning, Manassas, VA, USA), 100 mg/ml heparin (Catalog #67457037399, Mylan Institutional, Rockford, IL, USA) and 1% penicillin/streptomycin solution (Catalog #30-002-CI, Corning, Manassas, VA, USA). HULECs (ATCC, CRL-3244, Manassas, VA, USA) were cultured in MCDB 131 medium (Catalog #10372019, Thermo Fisher Scientific (Waltham, MA, USA) supplemented with Microvascular Endothelial Cell Growth Kit-BBE (Catalog #PCS-110-040, ATCC, Manassas, VA, USA) containing 0.2% bovine brain extract, 5 ng/mL rh EGF, 10 mM L-glutamine, 0.75 Unit/mL heparin sulfate, 1 μg/mL hydrocortisone, 50 μg/mL ascorbic acid and 5% FBS.

### Influenza A viral infection

For *in vitro* influenza A viral infection, as previously described (24), frozen stocks of mouse-adapted influenza A/Hong Kong/8/68 virus (H3N2) were diluted 1:1000 in 1x PBS and viral inocula (multiplicities of infection [MOI] of 0.1) were added to EC monolayer. For heat inactivation: influenza virus stocks were incubated for 30 min at 22.0, 35.0, 38.3, 43.7, 49.6, 55.6, 61.3, 66.7, and 70°C in a T-Gradient thermal cycler (Biometra, Göttingen, Germany) and subsequently stored at −80°C until *in vitro* infection (24, 25).

### Gene expression profiling

Gene expression profiling was performed as we have previously described (20). Briefly, HUVECs were transfected with either control siRNA (siCont) or MAGI1 siRNA (siMAGI1), and total RNA was isolated approximately 48 h post-transfection using RNeasy Plus Micro Kit (Cat# 74034, QIAGEN, Germantown, MD) with DNA digestion. Total RNA was then hybridized using a GeneChip Human Transcriptome Array 2.0 (Affymetrix, Santa Clara, CA). The array data was analyzed using the Transcriptome Analysis Console 3.0 (Affymetrix) followed by data normalization using Tukey's biweight average algorithm. Significance was determined using unpaired ANOVA (*P* < 0.05) as we have previously described (20). The differential expression data were analyzed using Ingenuity Pathway Analysis (IPA) (application build 377306M [2016-03-16] and content version 27216297 [2016-03-16]; QIAGEN). All molecular interactions and relationships were based on curated findings in the literature stored in the Ingenuity Knowledge Base (QIAGEN).

### siRNA-mediated MAGI1 depletion and transient MAGI1 overexpression

The siRNA sequence corresponding to nucleotides 843-857 of human MAGI1 coding sequence (5'-GGACCCUUCUCAGAAGUUCCCUCAA) (20) that specifically targets human MAGI1 for degradation was obtained from Sigma-Aldrich (Burlington, MA, USA). Authenticated siRNA sequence targeting human MX1 was obtained from Santa Cruz Biotechnology (Santa Cruz, Dallas, TX, USA). Non-target siRNA sequence (control) was obtained from Thermo Fisher Scientific (Waltham, MA, USA). siRNA-mediated MAGI1 depletion and transient MAGI1 overexpression were performed as we have previously described (20).

### RNA extracts and qRT-PCR

Studies were performed 48 h after siRNA transfection, as indicated in each corresponding figure. Total RNA was then extracted using the PureLink RNA mini-Kit (Thermo Fisher Scientific; Waltham, MA, USA). cDNAs were synthesized using the iScript cDNA synthesis Kit (Bio-Rad, Hercules, CA 94547, USA). Mixtures for qRT-PCR reactions (10 μL) contained cDNA synthesized from 20 ng of total RNA, 5 μ L of iQ SYBR Green Supermix (Bio-Rad, Hercules, CA 94547, USA), and 0.5 μM each forward and reverse primer. qRT-PCR reactions were carried out at 95°C for 3 min followed by 40 cycles of denaturation at 95°C for 10 s, 60°C for 15 s, and 72°C for 30 s. qRT-PCR data acquisition was carried out using the CFX Connect Real-Time PCR Detection System (Bio-Rad, Hercules, CA 94547, USA). The comparative C_t_ (^2−ΔΔCt^) method was used to relatively quantified changes in mRNA expression of samples, in which cycle threshold (Ct) values of target genes were normalized to that of the reference genes (26). All qRT-PCR primers were obtained from Sigma-Aldrich. The sequences of qRT-PCR primers were listed in the Supplementary Table 1.

### Protein extracts and immunoblotting

The cells were lysed on ice in RIPA buffer (50 mM Tris-HCl, pH 7.4, 150 mM NaCl, 1 mM ethylenediaminetetraacetic acid, 1% Nonidet P-40, 0.1% sodium dodecyl sulfate, 0.25% sodium deoxycholate) containing protease and phosphatase inhibitors (Sigma-Roche, Mannheim, Germany) (20). Cell lysates were briefly sonicated and then centrifuged at 16,000 xg for 10 min at 4°C to remove any debris. Cell extracts were mixed with SDS gel loading buffer and loaded on SDS-PAGE gels and separated proteins were transferred to nitrocellulose membranes. The membranes were probed with antibodies that specifically recognize proteins of interest. Some samples were analyzed by using the automated capillary electrophoresis Western analysis (Wes) system (ProteinSimple, San Jose, CA, USA) as we have performed previously (27).

### Antibodies and reagents

The following antibodies were purchased: MAGI1 (Catalog #M5691, Sigma-Aldrich, St. Louis, MO, USA), STAT1 (Catalog #ab103813, Abcam, Cambridge, MA, USA), MX1 (Catalog #sc-271024, Santa Cruz Biotechnology, Dallas, TX, USA), phopsho-STAT1 Y701 (Catalog #9167S, Cell Signaling, Danvers, MA, USA), IRF3 (Catalog #ab76409, Abcam, Cambridge, MA, USA), phopsho-IRF3 S396 (Catalog #29047, Cell Signaling, Danvers, MA, USA), GAPDH (Catalog #ab9484, Abcam, Cambridge, MA, USA), b-actin (Catalog #4970, Cell Signaling, Danvers, MA, USA), NP (Catalog #sc-80481, Santa Cruz, Biotechnology, Dallas, TX, USA), and M2 (Catalog #sc32238, Santa Cruz, Biotechnology, Dallas, TX, USA).

### OxLDL preparation

LDL at 0.2 mg/mL was incubated with 5 mM CuSO_4_ at 37°C for 24 h. The oxidation reaction was stopped by the addition of 20 mM EDTA. The preparations were concentrated by cone filtration and followed by dialyzed and sterilized by filtration through a 0.22 mm filter. The extent of oxidation of the LDL preparation was determined by measuring Thiobarbituric acid reactive substance (TBARS, Catalog #10009055; Cayman Chemicals, Ann Arbor, MI, USA). Another control LDL sample was processed in parallel, without CuSO_4_ addition. The results showed that OxLDL contained 14 nmol/mg protein and LDL contained 1 TBBARS/mg protein.

### Human IFN-β in cell culture supernates

ECs were transfected with either siCont or siMAGI1. Approximately after 48 h of transfection, conditioned medium was collected and levels of IFNβ were measured using the human IFN-β Quantikine ELISA kit (Catalog #DIFNB0, R&D Systems, MN, USA).

### Immunofluorescent staining

At the end of experiment, the cells were quickly washed twice with warm PBS, fixed with 4% paraformaldehyde in PBS for 15 min, and permeabilized with 0.2% Triton X-100 in PBS for 10 min. The cells were then incubated with a blocking solution (5% goat serum and 0.1% NP-40 in PBS) for 60 min at room temperature and incubated with the primary antibodies (IRF3 or NP) overnight at 4°C. Next, the cells were washed three times with PBS and incubated with fluorescently labeled secondary antibodies for 1 h at room temperature then were counterstained with 4′,6-diamidino-2-phenylindole (DAPI) to identify nuclei at room temperature. Imaging was done using an Olympus FV1200 multiphoton-confocal dual microscope (Olympus, Tokyo, Japan).

### Statistical analysis

First, we performed Shapiro-Wilk test for checking the normality of each group, and we performed an ordinary one-way ANOVA followed by Fisher's LSD testing for multiple group comparisons or unpaired student *t*-test with only the data that passed these normality tests. If the data did not pass the normality test, we performed Brown-Forsythe and Welch ANOVA or unpaired t-test with Welch's correction tests using the Prism software (GraphPad Software). *P-*values < 0.05 were considered statistically significant.

### Data availability

The microarray data and MAGI1 sequence were deposited in the NCBI's Gene Expression Omnibus database (accession GSE95066) and GenBank (accession KY651081), respectively. All other data, analytic methods, and study materials that support the findings of this study are available in the Data supplement or from the corresponding authors upon reasonable request.

## Results

### IAV infection increases MAGI1 expression and MAGI1 depletion suppresses IAV infection

**Figure 1:**
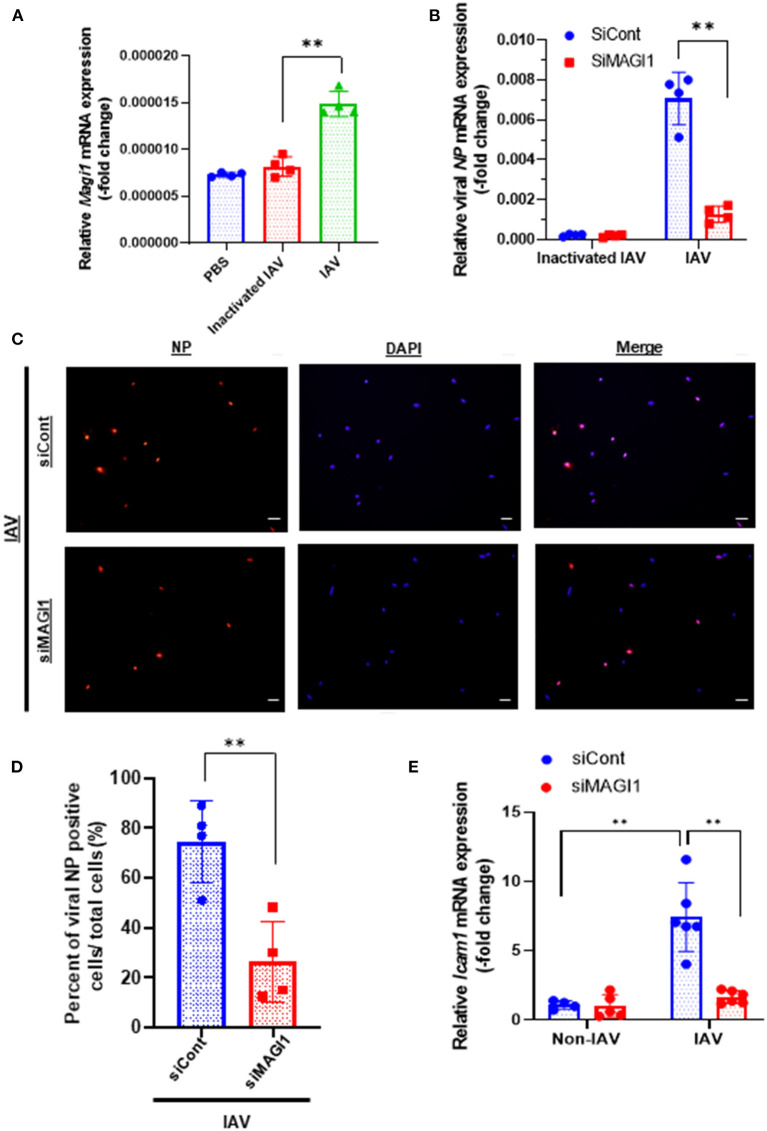
IAV infection increases MAGI1 expression and MAGI1 depletion suppresses IAV infection and inhibits EC inflammation. **(A)** HUVECs were infected with IAV, heat-inactivated virus, or treated with PBS for 24 h. Total RNA was extracted and qRT-PCR was performed. Relative changes in *Magi1* mRNA expression was calculated using the comparative C_t_ (^2−ΔΔCt^) method. Ct values of *Magi1* were normalized to that of *Gapdh* (26). Each group passed the Shapiro-Wilk normality test, then one-way ANOVA followed by Turkey's multiple comparisons test were performed using the Prism software (GraphPad Software). The graph shows mean ± SD (*n* = 4). **p < 0.01. **(B)** HUVECs were transfected with either siCont or siMAGI1. After 48 h of transfection, the cells were infected with IAV or heat-inactivated virus for 24 h. Total RNA was extracted then qRT-PCR was performed. Relative changes in the viral NP mRNA expression was calculated using the comparative C_t_ (^2−ΔΔCt^) method. Ct values of the viral NP were normalized to that of the 18S ribosomal RNA (26). Each group passed the Shapiro-Wilk normality test, then two-way ANOVA followed by Turkey's multiple comparisons test were performed using the Prism software (GraphPad Software). The graph shows mean ± SD (*n* = 3). **p < 0.01. **(C)** HUVECs were transfected with either siCont or siMAGI1. After 48 h of transfection, the cells were infected with IAV and immunofluorescence staining for the viral NP protein (red) and DAPI staining for the nucleus (blue) were performed. Scale bars: 20 μM. **(D)** We analyzed a total of 30–120 cells per 2–3 fields per dish to determine the percentage of NP positive cells in a blinded manner. Each group passed the Shapiro-Wilk normality test, then unpaired student *t*-test was performed using the Prism software (GraphPad Software). The graph shows mean ± SD (*n* = 4). **p < 0.01. **(E)** HUVECs were transfected with either siCont or siMAGI1. After 48 h of transfection, the cells were infected or not infected with IAV for 24 h. Total RNA was extracted, and qRT-PCR was performed. Relative changes in *Icam-1* mRNA expression was calculated using the comparative C_t_ (^2−ΔΔCt^) method. Ct values of *Icam-1* were normalized to that of *gapdh* (26). Each group passed the Shapiro-Wilk normality test, then two-way ANOVA followed by Turkey's multiple comparisons test was performed using the Prism software (GraphPad Software). The graph shows mean ± SD (*n* = 4–6). **p < 0.01.

To determine if MAGI1 expression in ECs in response to IAV infection promotes atherogenesis, we infected primary HUVECs with IAV. Given that the umbilical vein carries oxygenated blood, which is completely different from the systemic venous blood, HUVEC is the preferred *in vitro* cell culture system for determining the molecular mechanism of EC activation-induced atherogenesis (28). HUVECs infected with heat-inactivated virus was used as the mock control. After 24 h of infection, the cells were lysed, total RNA was extracted and analyzed by qRT-PCR to determine relative changes in *Magi1* mRNA expression levels (Figure 1A). Neither phosphate-buffered saline (PBS, vehicle control) nor the heat-inactivated virus (mock treatment control) had an effect, whereas the IAV infected cells increased *Magi1* mRNA expression level. We next knocked down MAGI1 by transfecting HUVECs with either the small interfering RNA (siRNA) that specifically targets *Magi1* mRNA for degradation (siMAGI1) or the siRNA negative control (siCont). After 48 h of siRNA transfection, the cells were infected with either IAV or mock control. After 24 h of virus infection, the cells were lysed, and total RNA was extracted and analyzed by qRT-PCR to determine relative changes in viral nucleoprotein (NP) mRNA expression levels. With the heat-inactivated IAV mock infection, there was no difference between siMAGI1 and siCont samples in the level of viral NP mRNA expression (Figure 1B). Conversely, infecting HUVECs transfected with siMAGI1 with live virus drastically downregulates the viral NP mRNA expression level relative to that of the siCont (Figure 1B). Studies of the cells themselves using immunofluorescence staining for the influenza NP protein showed similar results (Figures 1C,D).

Because MAGI1 induces EC activation (20), we investigated whether IAV activated ECs through upregulation of MAGI1 by determining relative changes in mRNA expression level of intercellular adhesion molecule 1 (ICAM-1), a marker of EC activation, using qRT-PCR in MAGI1-depleted HUVECs with or without virus infection. IAV infection increased *Icam-1* mRNA expression level and knocking down MAGI1 prevented this virus induced upregulation of *Icam-1* expression (Figure 1E). These results indicate that MAGI1 is essential to IAV infection, and in addition, mediates the activation of ECs by the virus. Interestingly, 24 h after virus infection, vascular cell adhesion molecule 1 (*Vcam-1*) mRNA expression was downregulated (Supplementary Figure 2).

**Figure 2:**
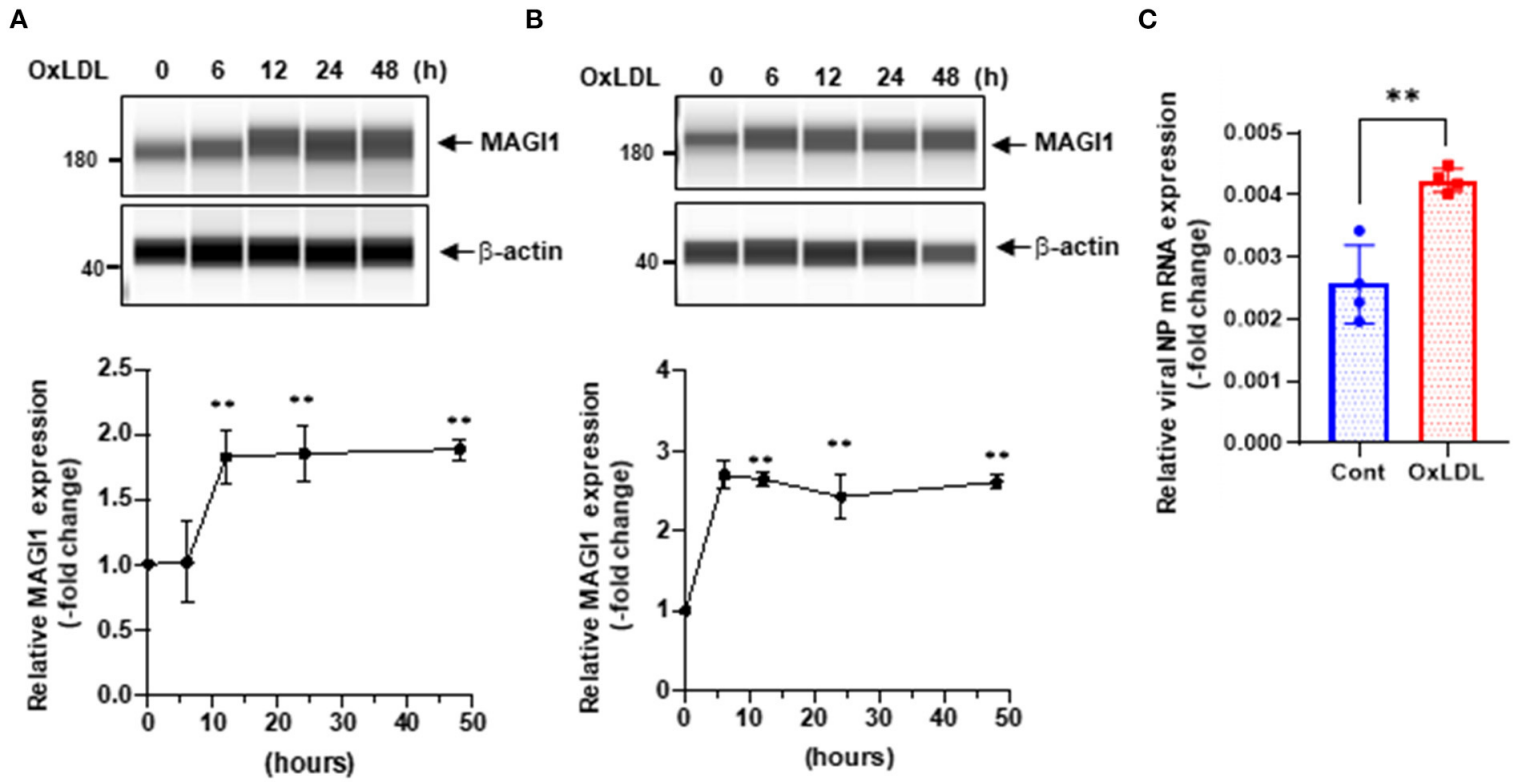
OxLDL increases MAGI1 expression and enhances IAV infection in ECs. **(A,B)** HUVECs **(A)** and HULECs **(B)** were pre-treated with OxLDL (10 μg/mL) for the indicated times. Upper: relative changes in MAGI1 protein expression were analyzed using the automated capillary electrophoresis Western analysis (Wes) system (ProteinSimple, San Jose, CA, USA) (27). Lower (densitometric quantification): relative changes in MAGI1 protein expression after OxLDL treatment. Fold increases are shown after normalization with β-actin at each time point. Quantification was performed using Image J. Each group passed the Shapiro-Wilk normality test, then one-way ANOVA followed by Turkey's multiple comparisons test was performed using the Prism software (GraphPad Software). The graph shows mean ± SD (*n* = 3). **P < 0.01. **(C)** HUVECs were pre-treated with OxLDL (10 μg/mL) for 24 h then infected with IAV for another 24 h. Total RNA was extracted then qRT-PCR was performed. Relative changes in the viral NP mRNA expression were calculated using the comparative C_t_ (^2−ΔΔCt^) method. Ct values of the viral NP were normalized to that of the 18S ribosomal RNA (26). Each group passed the Shapiro-Wilk normality test, then an unpaired student *t*-test was performed using the Prism software (GraphPad Software). The graph shows mean ± SD (*n* = 4). **p < 0.01.

We speculated that other pathological conditions in ECs also upregulate MAGI1 expression. IAV induces acute respiratory disease and the association between serum lipid levels and prognosis after severe acute respiratory syndrome coronavirus 2 (SARS-CoV-2) and influenza virus has been reported (29–34). Therefore, we investigated the role of MAGI1 in HUVECs and in human lung endothelial cells (HULECs) and found that OxLDL increased MAGI1 expression in both HUVECs and HULECs (Figures 2A,B). Furthermore, we also found that the pre-treatment of EC with OxLDL increased IAV infection (Figure 2C). Taken together, these data suggest a possible role for MAGI1 in IAV-associated cardiovascular and acute respiratory disease in dyslipidemia patients through driving virus infection.

### MAGI1 depletion upregulates IFN signaling

**Figure 3:**
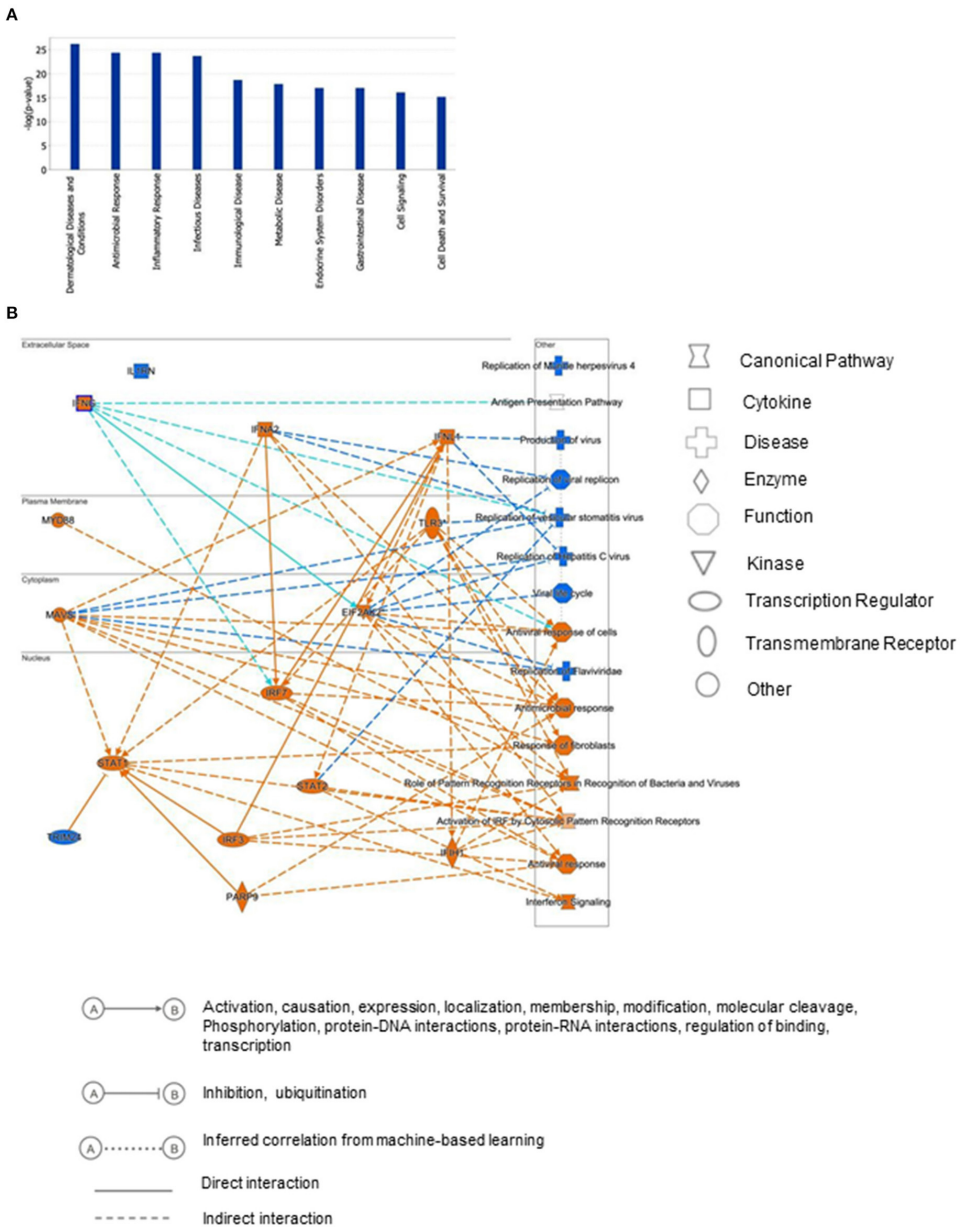
Differential gene expression profiles between siMAGI1 vs. siCont-treated ECs. **(A)** Statistical significance of the IPA-determined biological function and disease gene enrichment in siMAGI1-treated ECs calculated by the right-tailed Fisher exact test and presented as –log(*P*) value. A larger value on the *y*-axis represents greater significance. A total of 252 genes were differentially expressed in siMAGI1-transfected ECs compared to that in siCont-transfected ECs (*P* < 0.05; absolute fold change >2). The *p*-value, calculated by the Fischer's exact test, reflects the likelihood that the association among a set of genes in the data set and a related biological function is significant. **(B)** Graphical summary of IPA results showing significant predictor genes that are related to antiviral response.

To gain insights into the biological relevance of the reduced MAGI1 expression in ECs, we transfected HUVECs with either siCont or siMAGI1. Approximately 48 h after transfection, the cells were lysed, total RNA were extracted and transcriptionally profiled. A set of genes in the siMAGI1-treated ECs with Significant Differential Gene Expression (absolute fold-change >2, *p*-value < 0.05) was submitted to IPA (QIAGEN, Redwood City, CA) for core analysis. We identified 252 genes that were differentially expressed in siMAGI1-transfected ECs compared to that in siCont-transfected ECs. Using Fisher's Exact Test enrichment analysis for curated gene sets categorized by biological function and disease, we find that gene sets categorized as Dermatological-Disease and Conditions, Antimicrobial Response, Inflammatory Response, and Infectious Disease are the top four most significant groups (-log[P] >20, Figure 3A). The gene enrichment calculations and predicted biological gene effects, based upon directional expression changes and literature-substantiated causal relationships, indicated that the gene expression pattern will reduce viral infection in siMAGI1-transfected ECs compared to that in siCont-transfected ECs and that 45 of the 82 genes had the measurement direction consistent with decreased viral infection (*z-*score, −2.7; *P* = 5.6E-23) (Supplementary Table 2). The high *z-*score predicts that MAGI1 depletion will down-regulate the expression of genes belonging to the viral infection category.

Figure 3B is the graphical summary constructed by the algorithm based on machine learning techniques to prioritize and connect entities identified by IPA. It shows the relationship which may not yet be connected by findings in the QIAGEN Knowledge Graph and suggests that MAGI1 depletion induces strong antiviral responses and IFN signaling by increasing *Ifih1* (interferon-induced helicase C domain-containing protein 1), *Irf* (interferon regulatory factor) 3/7, *Parp9* (poly(ADP-ribose) polymerase family member 9), *Stat* (signal transducer and activator of transcription) 1/2, *Mavs* (mitochondrial antiviral signaling protein), *Eif2ak2* (eukaryotic translation initiation factor 2 alpha kinase), and *Ifng* (interferon gamma).

**Figure 4:**
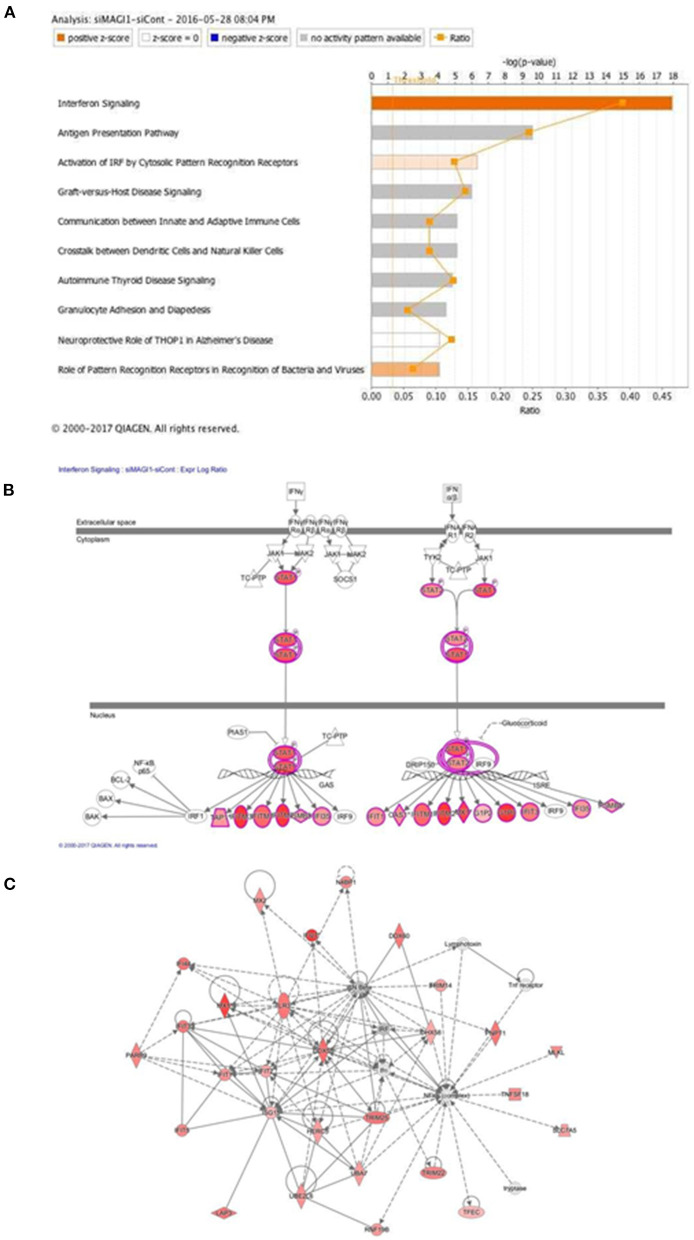
IFN signaling is the top-rank enriched IPA canonical pathway. **(A)** IPA-based identification of canonical pathways (–log[P] >5) associated with differentially expressed genes in HUVECs transfected with siMAGI1 vs. siCont. The bar chart indicates the –log(*P*) value of the significance of enrichment of each category. **(B)** Induction of IFN signaling in MAGI1-depleted ECs. Pathway models of IFN signaling in ECs based on data in the Ingenuity Knowledge Base are shown. Expression of genes in red is higher in siMAGI1-treated ECs compared to that in siCont-treated ECs. **(C)** Gene enrichment analyses (right-tailed Fisher exact test) according to the IPA downstream effects analysis identified Antimicrobial Response, Inflammatory Response, and Infectious Diseases as the highest scoring IPA interaction networks. Green and red symbols denote genes with lower and higher expression, respectively, in siMAGI1-treated ECs compared to that in siCont-treated ECs. The arrows with solid lines indicate direct (usually physical) interactions between two molecules in the direction of the arrow, Whereas, arrows with dashed lines denote indirect interactions (e.g., molecule/gene X affects molecule/gene Y). The abbreviations shown are defined in Supplementary Table 6.

The predicted biological gene expression affected by MAGI1 depletion is shown as heat maps (Supplementary Figure 1). The results show the unique and strong downregulation of infectious disease category (Supplementary Figure 1) including viral infection (*z-*score, −2.7; *P* = 5.6E-23, Supplementary Table 2), replication of virus (*z-*score, −4.1; *P* = 1.97E-24, Supplementary Table 3), infection of mammalia (*z-*score, −3.6; *P* = 9.1E-17, Supplementary Table 4), and production of virus (*z-*score, −3.1; *P* = 3.1E-07, Supplementary Table 5; Supplementary Figure 1B). We also find that IFN signaling is the top-ranked enriched IPA canonical pathway, which comprises of 36 genes, 14 of which are expressed at higher levels and thereby supporting the prediction that this pathway activation is increased in siMAGI1-transfected ECs compared to that in siCont-transfected ECs (*z-*score, 3.7; *P* = 1.1E-18) (Figures 4A,B; Supplementary Table 6). Following the IPA generated network, which optimizes the interconnectivity of differentially expressed genes under the constraint of the maximal network size, we carried out Fisher's Exact Test gene set function and disease enrichment analysis to predict biological functions that are expected to be affected by a given interaction network. The gene set function and disease enrichment of the top ranked interaction networks, based on the number of interconnected differentially expressed genes, were Antimicrobial Response, Inflammatory Response, and Infectious Diseases (differentially expressed genes, 28) (Figure 4C; Supplementary Table 7). As demonstrated in our network analysis (Figure 4C), although the expression of antiviral response-related genes, including *Mx1, Mx2*, and *Ddx58*, were upregulated in MAGI1-depleted ECs, NF-κB expression did not exceed the gene expression significance threshold.

**Figure 5:**
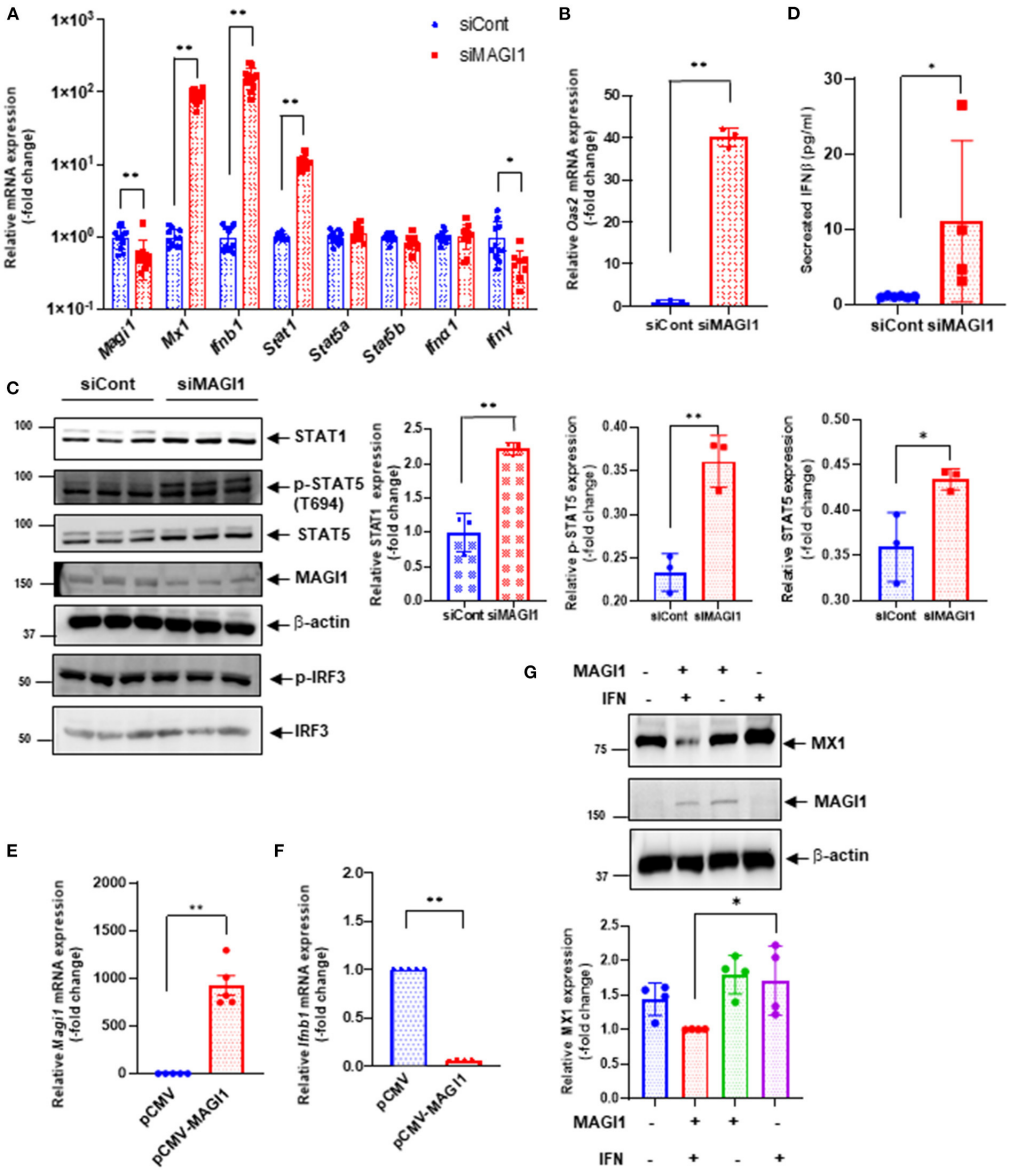
MAGI1 suppresses IFN signaling in ECs. **(A)** HUVECs were transfected with either siCont or siMAGI1. After 48 h of transfection, total RNA was extracted then qRT-PCR was performed. Relative changes in *Magi1, Mx1, Ifnb1, Ifna1, IfngI, Stat1, Stat5a, and Stat5b* expression were calculated using the comparative C_t_ (^2−ΔΔCt^) method. Ct values of each target gene were normalized to that of the *Gapdh* (26). Each group passed the Shapiro-Wilk normality test, then an unpaired student *t*-test was performed using the Prism software (GraphPad Software). The graph shows mean ± SD (*n* = 11). **p < 0.01, *p < 0.05. **(B)** HUVECs were transfected with either siCont or siMAGI1. After 48 h of transfection, total RNA was extracted then qRT-PCR was performed. Relative changes in *Oas2* mRNA expression were calculated using the comparative C_t_ (^2−ΔΔCt^) method. Ct values of *Oas2* were normalized to that of the *Gapdh* (26). Each group passed the Shapiro-Wilk normality test, then an unpaired student *t*-test was performed using the Prism software (GraphPad Software). The graph shows mean ± SD (*n* = 3). **p < 0.01. **(C)** HUVECs were transfected with either siCont or siMAGI1. After 48 h of transfection, cell lysates were analyzed by immunoblotting with each specific antibody as indicated. The graphs represent densitometry data from 3 independent gels, one of which is shown in the left panel. Each group passed the Shapiro-Wilk normality test, then an unpaired student *t*-test was performed using the Prism software (GraphPad Software). The graph shows mean ± SD, *n* = 3, **P < 0.01, and * *P* < 0.05. Uncropped figures were provided in the supplements. **(D)** HUVECs were transfected with either siCont or siMAGI1. After 48 h of transfection, conditioned medium was collected and levels of IFNβ were measured. Each group passed the Shapiro-Wilk normality test, then an unpaired student *t*-test was performed using the Prism software (GraphPad Software). The graph shows mean ± SD, *n* = 4–6, *P < 0.05. **(E,F)** HUVECs were transfected with either the pCMV-MAGI1 or the pCMV-2B backbone construct (control) (20). After 24 h of transfection, total RNA was extracted then qRT-PCR was performed. Relative changes in *Magi1* **(E)** and *Ifnb1* **(F)** mRNA expression were calculated using the comparative C_t_ (^2−ΔΔCt^) method. Ct values of each target gene were normalized to that of the *gapdh* (26). At least, one of the groups did not pass the Shapiro-Wilk normality test, we performed an unpaired *t*-test with Welch's correction using the Prism software (GraphPad Software). The graph shows mean ± SD, *n* = 5, **P < 0.01. **(G)** HUVECs were transfected with either the pCMV-MAGI1 or the pCMV-2B backbone construct (control) (20). After 24 h of transfection, the cells were treated with IFN (200 U/mL) for 6 h, and immunoblotting was performed with each specific antibodies as indicated. The graph (lower) represents densitometry data from 4 independent gels, one of which is shown in the top panel. Each group passed the Shapiro-Wilk normality test, then one-way ANOVA followed by Turkey's multiple comparisons test was performed using the Prism software (GraphPad Software). The graph shows mean ± SD (*n* = 4). *p < 0.05.

To verify key microarray results, we performed qRT-PCR using HUVECs transfected with siCont or siMAGI1 and evaluated relative changes in mRNA expression level of *Magi1, Mx1, Ifnb1, Ifna1, Ifng, Stat1, Stat5a*, and *Stat5b*. Knocking down MAGI1 increases mRNA expression level of *Ifnb1, Mx1* and *Stat1*, but decreases mRNA expression level of *Ifng*. MAGI1 depletion did not alter mRNA levels of *Ifna1, Stat5a*, and *Stat5b* (Figure 5A). To determine the anti-viral effects by MAGI1 depletion, we also evaluated *Oas2*, another anti-viral molecule that can cause viral RNA degradation and inhibition of viral replication (35) and find that depletion of MAGI1 by siRNA upregulated *Oas2* mRNA expression (Figure 5B). Although the goal of this study is to investigate the role of MAGI1 in IAV-associated cardiovascular diseases, we also treated HULECs with siCont or siMAGI1 and quantified *Ifnb1* mRNA expression and observed a clear increase in *Ifnb1* mRNA expression (Supplementary Figures 2B,C), indicating that the yin-yang form of correlation between *Magi1* and *Ifnb1* expressions is not specific to HUVECs.

We also confirmed key microarray results using immunoblotting to detect protein expression level. STAT1 protein expression was augmented by MAGI1 depletion (Figure 5C). Total STAT5 activity (p-STAT5 T694 represents total STAT5 activity including total STAT5 expression) was increased by siMAGI1 transfection (Figure 5C). We have also shown that the level of secreted IFNb in conditioned medium from siMAGI1-transfected ECs was increased compared to that from siCont-transfected ECs (Figure 5D). These data suggest that MAGI1 depletion differentially affects IFN signaling both in mRNA and protein levels.

To clarify the role of MAGI1 in IFN signaling, we performed two types of overexpression studies. First, we overexpressed MAGI1 and measured the relative changes in *Ifnb1* mRNA expressions. In ECs overexpressing MAGI1, *Ifnb1* mRNA expression levels were decreased (Figures 5E,F). Next, we determined if MAGI1 overexpression altered MX1 expression and observed little change (data not shown). Therefore, we stimulated ECs overexpressing MAGI1 with a low dose of IFN (200 U/mL), and observed a decreased MX1 expression (Figure 5G). It is unclear why *Ifnb1* mRNA and MX1 protein expression respond differently to MAGI1 overexpression; however, it is possible that IFN-mediated signaling such as p90RSK activation may potentiate the inhibitory effects of MAGI1 on MX1 expression through promoting MAGI1 post-translational modification (20). These data support a role for MAGI1 in promoting viral infection in ECs.

### Knocking down MAGI1 suppresses IAV infection partially through increasing MX1 expression

**Figure 6:**
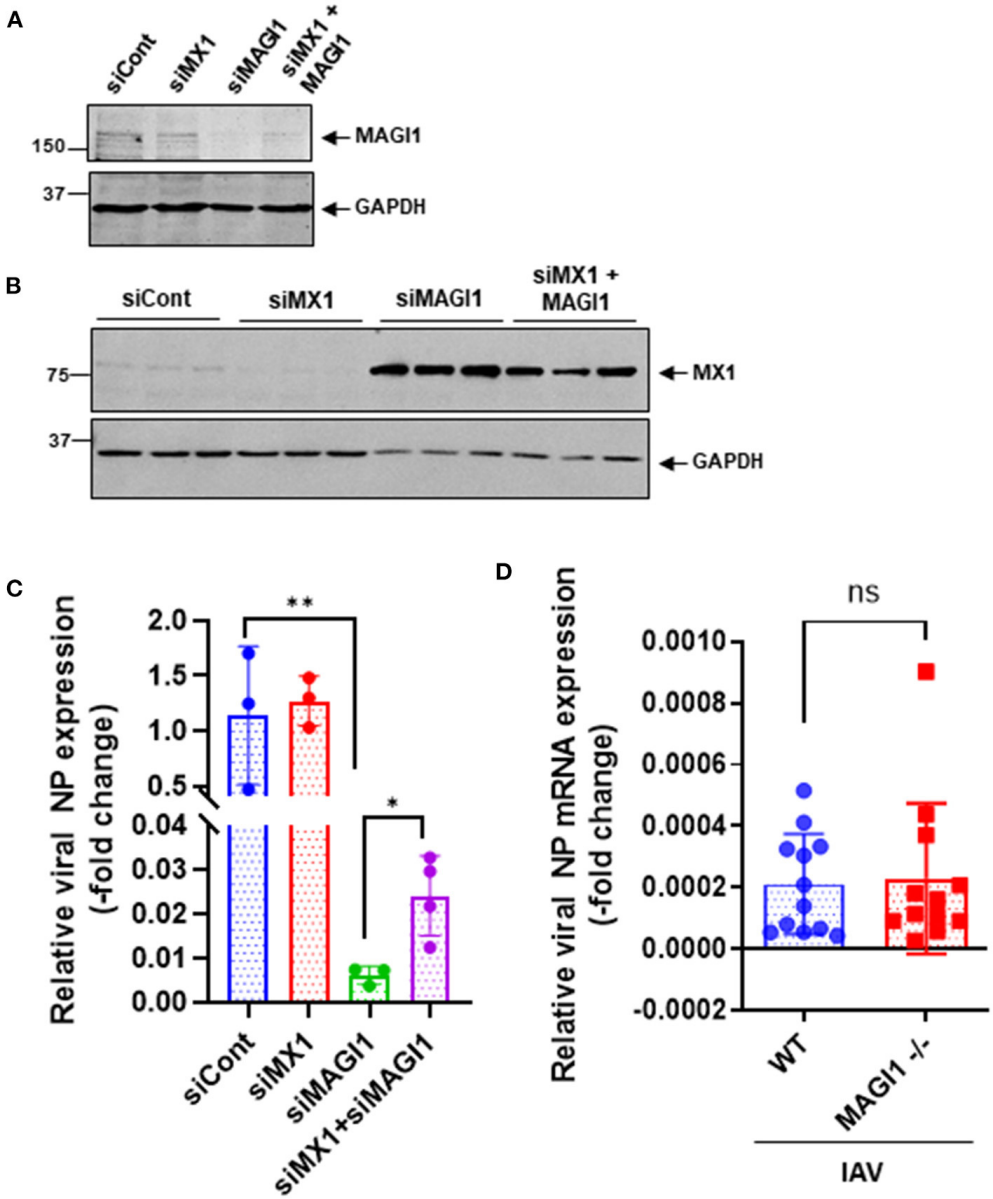
MX1 depletion reverses MAGI1 depletion-mediated suppression of IAV infection. **(A,B)** HUVECs were transfected with either siCont, siMX1, siMAGI1, or siMX1+siMAGI1. After 48 h of transfection, cell lysates were analyzed by immunoblotting with each specific antibody as indicated. Gels are representative of 3 independent experiments. **(C)** HUVECs were transfected with either siCont, siMX1, siMAGI1, or siMX1+siMAGI1. After 48 h of transfection, the cells were infected with IAV for 24 h. Total RNA was extracted then qRT-PCR was performed. Relative changes in the viral NP mRNA expression were calculated using the comparative C_t_ (^2−ΔΔCt^) method. Ct values of the viral NP were normalized to that of the 18S ribosomal RNA (26). Each group passed the Shapiro-Wilk normality test, then one-way ANOVA followed by Turkey's multiple comparisons test was performed using the Prism software (GraphPad Software). The graph shows mean ± SD (*n* = 3-4). **p < 0.01, *p < 0.05. **(D)** Mouse lung ECs were isolated from *Magi1*^−/−^ and littermate wild type (WT) control mice and cultured. The cells were infected with IAV for 24h. Total RNA was extracted then qRT-PCR was performed. Relative changes in the viral NP mRNA expression were calculated using the comparative C_t_ (^2−ΔΔCt^) method. Ct values of the viral NP were normalized to that of the 18S ribosomal RNA (26). Each group passed the Shapiro-Wilk normality test, then an unpaired student *t*-test was performed using the Prism software (GraphPad Software). The graph shows mean ± SD, n =11. NS: not significant.

**Figure 7:**
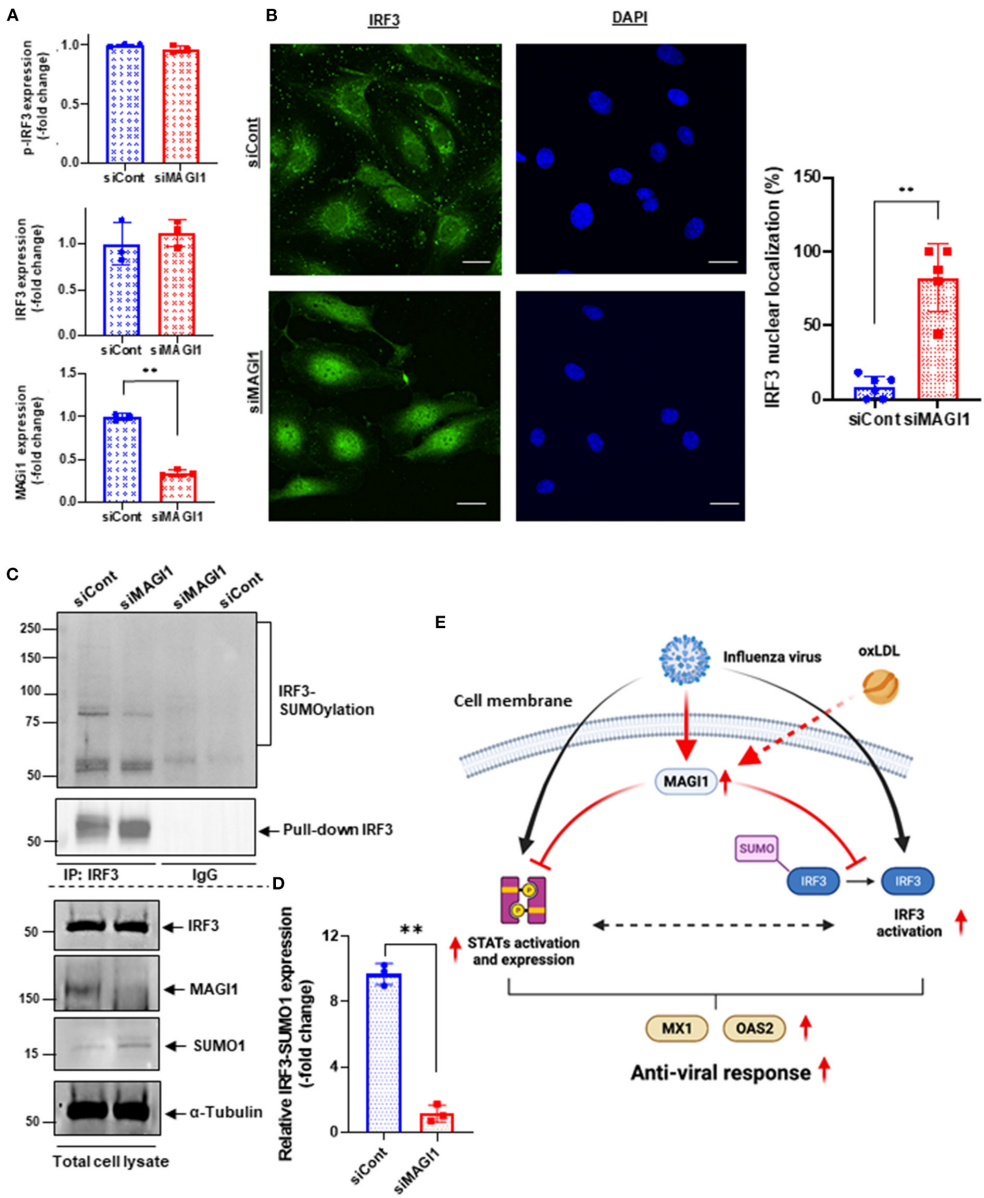
MAGI1 depletion promotes IRF3 nuclear translocation and de-SUMOylation without affecting IRF3 S396 phosphorylation. **(A)** HUVECs were transfected with either siCont or siMAGI1. After 48 h of transfection, cell lysates were analyzed by immunoblotting with each specific antibody as indicated. The graphs represent densitometry data from 3 independent gels, one of which is shown in Figure 5C. Each group passed the Shapiro-Wilk normality test, then an unpaired student *t*-test was performed using the Prism software (GraphPad Software). The graphs show mean ± SD, *n* = 3, **P < 0.01. **(B)** HUVECs were transfected with either siCont or siMAGI1. After 48 h of transfection, immunofluorescence staining for IRF3 protein (green) and DAPI staining for the nuclei (blue) were performed. Scale bars: 20 μM. Robust IRF3 nuclear translocation noted in siMAGI1-transfected cells. The percentages of cells with nuclear staining were quantified by counting the cells with the mean intensity of IRF3 staining in the nucleus ≥ mean intensity of IRF3 cytoplasmic staining by each dish (*n* = 5–6) in a blinded manner. **(C)** HUVECs were transfected with either siCont or siMAGI1. After 48 h of transfection, cell lysates were immuno-precipitated IRF3 with the antibody that specifically recognizes this protein then immunoblotting with the antibody that recognizes SUMO1 to detect IRF3 SUMOylation (20). IgG antibody was used as a negative control. Total cell lysates were immunoblotted with each specific antibody as indicated. **(D)** The graphs represent densitometry data from 3 independent gels, one of which is shown in shown in **(C)**. Median intensities of IRF3 SUMOylation were calculated after subtracting the background, which is median intensities of IgG control. Each group passed the Shapiro-Wilk normality test, then an unpaired student *t*-test with Welch's correction was performed using the Prism software (GraphPad Software). The graph shows mean ± SD, *n* = 3, **P < 0.01. **(E)** The scheme depicts the relationship between MAGI1 and IFN signaling. IAV infection induces anti-viral responses including STATs and IRF3 activation and subsequent MX1 and OAS2 induction. For efficient infection of IAV to ECs, IAV induces MAGI1 expression, resulting in inhibition of anti-viral responses by inhibiting STATs and IRF3 activation. Especially, MAGI1 inhibits IRF3 de-SUMOylation and subsequent IRF3 activation. Importantly, OxLDL also upregulates MAGI1 expression and promotes IAV infection, which may explain the relationship between hypercholesterolemia and IAV infection in patients. The scheme was generated using BioRender.

Since MX1 exerts broad antiviral activity against several strains of viruses including IAV (36–38), we tested whether the MAGI1 depletion-induced virus suppression could be due to increased MX1 expression. HUVECs were transfected with siCont, siMX1, siMAGI1, or siMX1+siMAGI1 and then infected with IAV. We extracted total RNA from these cells and performed qRT-PCR to detect relative changes in viral NP mRNA expressions. Cell lysates were also immunoblotted for MX1 protein expression. Knocking down MAGI1 increased MX1 expression. However, depletion of MX1 exerted no effect on MAGI1 expression (Figures 6A,B). Since we also noted the reduced MX1 expression by MAGI1 overexpression only occurs under IFN stimulation (Figure 5G), these data have suggested that MAGI1 is an upstream signaling event for regulating MX1 expression in ECs. The depletion of MAGI1 by siMAGI1 increased MX1 expression (Figure 6B), and the partial significant inhibition of MX1 expression after siMX1 transfection was due to the incomplete transfection efficiency by siMX1. We also observed that siMX1 reversed the anti-viral effects induced by siMAGI1, suggesting that siMAGI1-mediated induction of MX1 plays a meaningful role in siMAGI1-mediated anti-viral response. However, siMAGI1 leads to the inhibition of influenza infection (Figure 6C), which was in part due to the induction of MX1. In fact, in addition to MX1, we also found that the depletion of MAGI1 by siMAGI1 upregulated *Oas2* mRNA expression (Figure 5B). Furthermore, we also found the upregulation of *Ifnb1* (Figures 5A,B) and *Stat1* (Figures 5A,C) expression, STAT5 activation (Figure 5C), and IRF3 nuclear translocation (Figure 7B) which played a significant role in developing anti-viral response (Figure 7E) (39–42). Therefore, siMAGI1 can induce anti-viral effects not only through MX1 but also by induction of other anti-viral molecules (Figure 7E).

The *Mx1* gene in C57BL/6 mice contains a large deletion, which creates the MX1 null phenotype, a condition that increases susceptibility of this mouse strain to IAV (43, 44). We took advantage of this fact (i.e., *Mx1* null condition in C57BL/6 mice) to further determine if MAGI1 depletion in mice lacking MX1 still inhibits IAV infection. Mouse ECs were isolated from the lungs of *Magi1*^−/−^ and WT littermate control mice and cultured to confluence on gelatin-coated plates. The cells were then infected with virus for 24 h. Total mRNA was extracted from the infected cells, and relative changes in viral NP mRNA expression levels were determined by qRT-PCR (Figure 6D). No difference in NP mRNA expression was found between WT and *Magi*^−/−^ mouse ECs. Whereas, we showed that NP mRNA burden is correlated with viral burden and infectivity (24), these results indicate that MX1 promotes the inhibition of virus replication caused by MAGI1 depletion.

### Knocking down MAGI1 promotes IRF3 nuclear translocation and de-SUMOylation without affecting IRF3 phosphorylation

MAGI1 depletion in human lung epithelial cells increases phosphorylation of IRF3, an upstream transcriptional regulator of IFN genes, at S396, which triggers IRF3 translocation into the nucleus and activation IFN-β signaling (45, 46). Following activation in the cytoplasm, IRF3 translocate into the nucleus and promotes the transcription of IFN-α and -β and IFN-stimulated genes by binding to the interferon-stimulated response element (ISRE) (47, 48). However, it is still not clear if ECs respond in the same manner. To address this question, we treated HUVECs with either siMAGI1 or siCont and detected IRF3 S396 phosphorylation and nuclear translocation. According to immunoblot analysis, similar levels of IRF3 phosphorylation at S396 was observed in control and MAGI1-depleted cells (Figures 5C, 7A,C). By contrast, IRF3 nuclear translocation was clearly observed in MAGI1-depleted HUVECs while the cytoplasmic staining was prevalent in cells treated with siCont (Figure 7B), suggesting that IRF3 nuclear translocation is involved in siMAGI1-mediated upregulation of IFN expression. Kubota et al. showed that virus infection enhances IRF3 SUMOylation, thereby attenuating IFN production (49). This finding has led us to investigate the potential role of MAGI1 on IRF3 SUMOylation. After 48 h of either siMAGI1 or siCont transfection, we lysed the cells and immuno-precipitated IRF3 with the antibody that specifically recognizes this protein. IgG antibody was used as a negative control. We then utilized immunoblotting to assess SUMOylated IRF3 using the antibody that recognizes only SUMO1. We found a significant decrease in IRF3 SUMOylation by MAGI1 depletion (Figures 7C,D), suggesting the upregulation of IRF3 transcriptional activity as described previously (49). These data suggest that MAGI1 depletion increased IRF3 transcriptional activity *via* downregulated IRF3 SUMOylation and promoted IRF3 nuclear translocation, which may be independent of IRF3 phosphorylation (Figure 7E).

## Discussion

In this study, we find that IAV infection and OxLDL pre-treatment upregulate MAGI1 expression in ECs. Influenza replication is increased by OxLDL pre-treatment, Whereas, MAGI1 depletion decreased virus replication, suggesting the crucial role of IAV-induced MAGI1 expression in promoting IAV infection in hypercholesterolemia patients. In MAGI1-depleted ECs, we also noted heightened anti-viral events such as upregulation of IFN signaling and response-related gene expression including MX1 and OAS2. Lastly, we find that MAGI1 depletion inhibits IRF3 SUMOylation, which subsequently induces IRF3 activation. Our results suggest that IAV and/or OxLDL-induced MAGI1 expression accelerates virus infection in ECs and that MAGI1 depletion effectively inhibits this process. The data collectively provides insights into the role of MAGI1 in influenza infection as well as associated cardiovascular disease observed in dyslipidemia patients (11–15).

This study is the first to show that IAV infection increases MAGI1 expression in cultured ECs and that MAGI1 depletion suppresses IAV replication by increasing MX1 expression, a process that appears to induce IFN production. Indeed, others have shown that increased MX1 expression induces IFN-β production (46). The consensus PD*Z-*binding motif ESEV (glu-ser-glu-val) (PBM) in NS1 protein of IAV binds the PDZ domain of various proteins. MAGI1 has six PDZ domains, thus, it is possible that the binding between the virus NS1 PBM and MAGI1 plays a major role in viral infection and replication (48, 50). Since we find that virus infection upregulated MAGI1 expression, this increased MAGI1 expression provides a favorable environment for virus to replicate and thereby forming a positive feedback loop. Thus, MAGI1 depletion should break this cycle and suppress virus infection and replication. Our results have supported this model. In this study, we have also shown that knocking down MX1 impairs MAGI1 depletion-mediated IAV suppression. Moreover, the impaired virus infection caused by MAGI1 depletion was not detected in cultured lung ECs derived from C57BL/6 mice that are known to carry a truncated *Mx1* gene, which results in the MX1 null phenotype. The anti-viral effect of MX1 *in vivo* was clearly demonstrated using mouse models with and without MX1 expression (51). Together, these results suggest that MAGI1 depletion mediates the suppression of IAV infection, at least in part, by increasing MX1 expression. We also find upregulation of a broad range of IFN signaling network-related molecules (Figure 4) including the increase of STAT1 and *Oas2* expression and STAT5 activity (Figure 5), revealing how the depletion of MAGI1 can induce a strong anti-viral response (Figure 7E).

IRF3, a key transcriptional regulator of type I interferon, regulates the transcription of IFN-β and IFN-stimulated genes by binding the ISRE in their promoters. Upon viral infection, IRF3 in the cytoplasm of the infected cells is phosphorylated by the kinases TBK1 and IKKε, and undergoes a conformational change and homo-dimerization, which permits its translocation to the nucleus where it binds the ISRE of target genes (46, 47). Knocking down MAGI1 specifically in lung epithelial cells causes IRF3 S396 phosphorylation and increased IFN production (46). Interestingly, although we found a clear IRF3 nuclear translocation after MAGI1 depletion, we did not detect significant increases of IRF3 phosphorylation under the same condition (Figures 7A,B). This led us to investigate other types of IRF3 post-translational modifications, and we found a significant decrease of IRF3 SUMOylation induced by MAGI1 depletion (Figures 7C,D). Since the activation of IRF3 by attenuating IRF3 SUMOylation has been reported (49), the decrease of IRF3 SUMOylation by siMAGI1 transfection can induce IRF3 nuclear translocation and transcriptional activity. Of note, we have reported that the similar effect of p53 SUMOylation in ECs cultured under disturbed flow leading to p53 nuclear export (52). Therefore, both p53 and IRF3 SUMOylation may be important to retrain these proteins in the cytoplasm, and after losing this modification, p53 and IRF3 translocate to the nucleus. Furthermore, we have previously reported that de-SUMOylation enzyme of sentrin/SUMO-specific protease 2 (SENP2) regulates MAGI1 SUMOylation and de-SUMOylation of MAGI1 induced by SENP2 promotes MAGI1 nuclear translocation and subsequent EC activation (20). Therefore, MAGI1 may inhibit IRF3 SUMOylation by modulating SENP2 function. Future studies will be necessary to clarify these issues.

EC activation is a major cause of cardiovascular disease. Our study revealed upregulation of ICAM-1 expression in IAV-infected ECs, indicating that virus infection activates ECs, and increasing the risk of cardiovascular dysfunction in influenza patients. In our recent study, we have shown that MAGI1 plays a major role in atherogenesis by inducing pro-inflammatory signaling in ECs (20). The role of MAGI1 in EC activation was confirmed by knock down studies, in which the virus induced ICAM-1 elevation was strongly inhibited. Together, these results show that MAGI1 could be the molecular link between IAV infection and cardiovascular disease. Furthermore, our current study shows that MAGI1 expression is upregulated under a pro-atherogenic, physiological perturbation i.e., OxLDL treatment (Figures 2A,B). More importantly, the pre-treatment of OxLDL upregulates virus infection in ECs (Figure 7C), suggesting that the induction of MAGI1 by OxLDL increases EC activation and accelerates virus infection by inhibiting IAV-mediated anti-viral responses (Figure 7E). It has been reported that IL-1β stimulation induces VCAM-1 expression rapidly and temporally although sustaining ICAM-1 expression over 24–72 h (53). Therefore, the inhibition of VCAM-1 expression in our study may be due to a counter-response to the initial increase of VCAM-1 expression induced by influenza A.

In addition to MAGI1, which has six PDZ domains, there are other PDZ domain-containing proteins—Dlg1, MAGI2, MAGI3, Scribble, Lin7C, PDLIM2 and PSD-95 - are potential targets of the NS1 protein of IAV that has the consensus PD*Z-*binding motif, ESEV (glu-ser-glu-val) (PBM). Indeed, PBM association with Scribble and Dlg1 promotes virus infection (48). Our study shows that MAGI1 also promotes virus infection, presumably *via* its interaction with PBM. Since MAGI1 depletion robustly inhibits IAV infection, MAGI1's role in supporting virus infection is larger than that of Scribble and Dlg1.

In conclusion, we have found that MAGI1 is involved in IAV infection of ECs. Previously, we have reported the crucial role of MAGI1 in EC activation and subsequent atherogenesis (20). In the current report, we found that IAV infection increases MAGI1 expression and enhances virus infection by inhibiting various anti-viral responses including STATs and IRF3 activation and induction of MX1 and OAS2 (Figure 7E). We also find that MAGI1 inhibits IRF3 de-SUMOylation, resulting in inhibiting IRF3 activation. These data suggest the crucial role of MAGI1 in inhibiting IAV-mediated anti-viral responses, and the induction of MAGI1 by OxLDL and IAV infection itself can accelerate further virus infection and promote severe EC activation (Figure 7E). Thus, MAGI1 as a promotor of both EC activation and virus infection, is a potential therapeutic target for influenza virus infection.

## Data availability statement

The datasets presented in this study can be found in online repositories. The microarray data and MAGI1 sequence were deposited in the NCBI's Gene Expression Omnibus database (accession GSE95066) and GenBank (accession KY651081), respectively.

## Ethics statement

The animal study was reviewed and approved by the Institutional Care and Use Committees of the Texas A&M Institute of Biosciences and Technology and The University of Texas MD Anderson Cancer Center.

## Author contributions

YinW performed experiments, analyzed data, and drafted manuscript. J-iA planned and oversaw the project, funded research, performed data analysis, and wrote the manuscript. KC, YongW, HV, LR, and FG performed experiments. MI, VS, MN, KK, L-LL, and TT supported the experiments and interpretation of the data. EO-D, JC, SK, and KF contributed to the interpretation of the data and edited the manuscript. SE and N-TL planned and generated the study design, obtained funding, interpreted data, and wrote the manuscript. All authors contributed to the article and approved the submitted version.

## Funding

This study was partially supported by funding's from the National Institutes of Health (NIH) to J-iA, N-TL, and JC (HL149303), J-iA (AI156921), JC (HL148338 and HL157790), N-TL (HL-134740), and SE (HL144805).

## Conflict of interest

The authors declare that the research was conducted in the absence of any commercial or financial relationships that could be construed as a potential conflict of interest.

## Publisher's note

All claims expressed in this article are solely those of the authors and do not necessarily represent those of their affiliated organizations, or those of the publisher, the editors and the reviewers. Any product that may be evaluated in this article, or claim that may be made by its manufacturer, is not guaranteed or endorsed by the publisher.

## Supplementary material

Supplementary Figure 1 Heat maps based on Ingenuity Downstream Effects Analysis (Diseases and Functions analysis). The maps show the functional hierarchy for differential gene expression in siMAGI1-transfected HUVECs with the significance and predicted activity state of gene sets identified by the Ingenuity Knowledge Base. The color-coded scales above both heat maps reflect the direction of change for each function based on activation *z-*scores (orange, upregulation; blue, downregulation; white, *z-*score of 0; gray, not available). **(A)** The level 1 squares in this heat map (high-level functional categories) indicate the functions predicted to increase or decrease with MAGI1 depletion. The size of each box reflects the *P*-value (the larger the box, the smaller the *P*-value). The *P*-values for the Infectious Disease categories are within the six highest category. The *z-*score for the Infectious Disease category is predicted to decrease with MAGI1 depletion. **(B)** The hierarchical heat map of the level 1 category Infectious Disease.

Supplementary Figure 2 *Magi1, Vcam1, and Ifn*β*1* mRNA expression. **(A)** HUVECs were transfected with either siCont or siMAGI1. Approximately after 48 h of transfection, the cells were infected or not infected with IAV for 24 h. Total RNA was extracted then qRT-PCR was performed. Relative changes in *Vcam1* mRNA expression were calculated using the comparative C_t_ (^2−ΔΔCt^) method. Ct values of *Vcam1* mRNA expression were normalized to that of *Gapdh* (26). Each group passed the Shapiro-Wilk normality test, then two-way ANOVA followed by Turkey's multiple comparisons test was performed using the Prism software (GraphPad Software). The graph shows mean ± SD (*n* = 3). ^**^*p* < 0.01. **(B,C)** HULECs were transfected with either siCont or siMAGI1. Approximately after 48 h of transfection, total RNA was extracted then qRT-PCR was performed. Relative changes in *Magi1* **(B)** and *Ifnb1* **(C)** mRNA expression were calculated using the comparative C_t_ (^2−ΔΔCt^) method. Ct values of each target gene were normalized to that of β*-actin* (26). At least, one of the groups did not pass the Shapiro-Wilk normality test, we performed an unpaired *t*-test with Welch's correction using the Prism software (GraphPad Software). The graph shows mean ± SD, *n* = 3, ^**^*P* < 0.01.

Supplementary Table 1 Oligonucleotide sequences.

Supplementary Table 2 Viral infection.

Supplementary Table 3 Replication of virus.

Supplementary Table 4 Infection of mammalia.

Supplementary Table 5 Production of virus.

Supplementary Table 6 Interferon signaling.

Supplementary Table 7 Antimicrobial response, inflammatory response, and infectious diseases.

## Abbreviations

DAPI, 4′: 6-diamidino-2-phenylindole
ECs: endothelial cells
GAPDH: glyceraldehyde-3-phosphate dehydrogenase
HULECs: human lung microvascular ECs
HUVECs: human umbilical vein ECs
IAV: influenza A virus
ICAM-1: intercellular adhesion molecule 1
IFN: interferon
IFIH1: interferon-induced helicase C domain-containing protein 1
IPA: ingenuity pathway analysis
IRF3: interferon regulatory factor 3
ISG: IFN-stimulated genes
ISRE: interferon-stimulated response element
M2: matrix protein 2
MAGI1: membrane-associated guanylate kinase with inverted domain structure-1
MAVS: mitochondrial antiviral signaling protein
MOI: multiplicities of infection
MX1: myxovirus resistance protein 1
OAS2: 2′-5′-oligoadenylate synthetase 2
PARP9: poly(ADP-ribose) polymerase family member 9
PBM: PDZ binding motif
PBS: phosphate buffered saline
NP: nucleoprotein
OxLDL: oxidized low-density lipoprotein
PDZ: PSD95/DiscLarge/ZO-1
PTEN: phosphatase and tension homolog
qRT-PCR: quantitative reverse-transcriptase polymerase chain reaction
RIPA: radioimmunoprecipitation assay buffer
SDS: sodium dodecyl sulfate
SDS-PAGE: SDS polyacrylamide gel electrophoresis
STAT1/2: signal transducer and activator of transcription $\frac{1}{2}$
siRNA: small interfering RNA
VCAM-1: vascular cell adhesion molecule 1.
