# Supplementary figures and images for: MAGI1 inhibits interferon signaling to promote influenza A infection
Source: Front Cardiovasc Med. 2022 Aug 23;9:791143. doi: 10.3389/fcvm.2022.791143 (PMC9445416; doi:10.3389/fcvm.2022.791143)

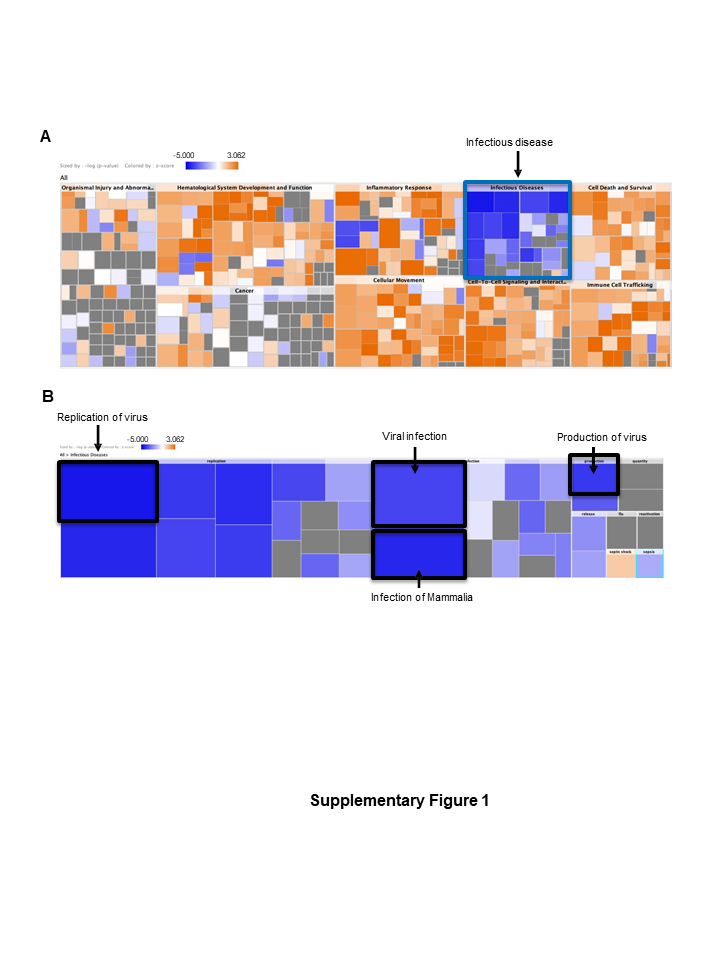

Supplement: Supplementary Figure 1 — Heat maps based on Ingenuity Downstream Effects Analysis (Diseases and Functions analysis). The maps show the functional hierarchy for differential gene expression in siMAGI1-transfected HUVECs with the significance and predicted activity state of gene sets identified by the Ingenuity Knowledge Base. The color-coded scales above both heat maps reflect the direction of change for each function based on activation z-scores (orange, upregulation; blue, downregulation; white, z-score of 0; gray, not available). (A) The level 1 squares in this heat map (high-level functional categories) indicate the functions predicted to increase or decrease with MAGI1 depletion. The size of each box reflects the P-value (the larger the box, the smaller the P-value). The P-values for the Infectious Disease categories are within the six highest category. The z-score for the Infectious Disease category is predicted to decrease with MAGI1 depletion. (B) The hierarchical heat map of the level 1 category Infectious Disease. [file Data_Sheet_1.zip › Supplementary Figure1.tif]

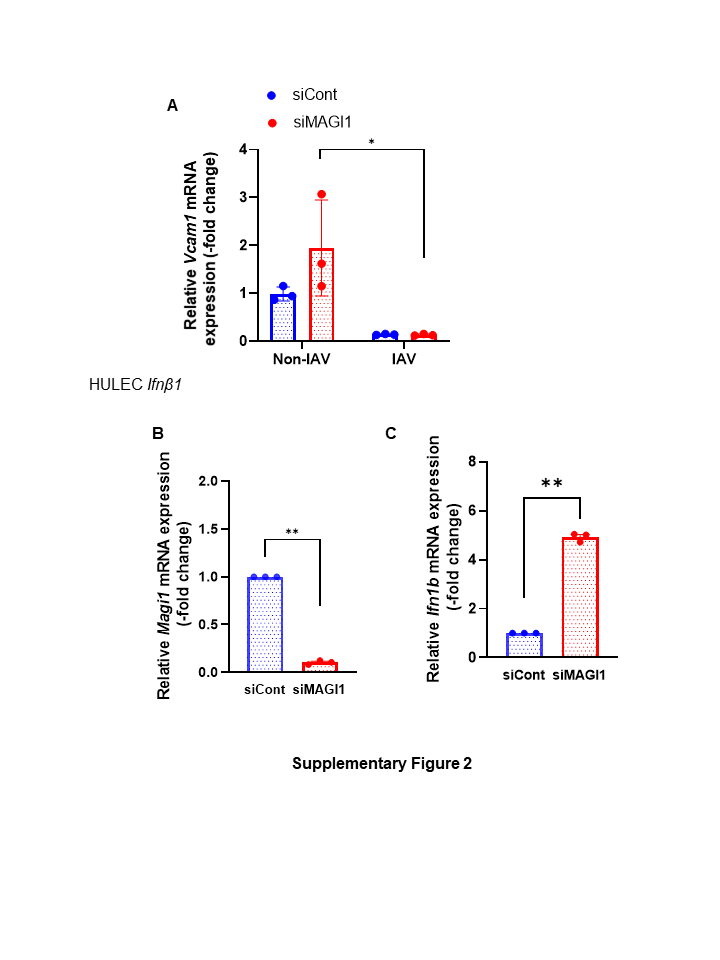

Supplement: Supplementary Figure 1 — Heat maps based on Ingenuity Downstream Effects Analysis (Diseases and Functions analysis). The maps show the functional hierarchy for differential gene expression in siMAGI1-transfected HUVECs with the significance and predicted activity state of gene sets identified by the Ingenuity Knowledge Base. The color-coded scales above both heat maps reflect the direction of change for each function based on activation z-scores (orange, upregulation; blue, downregulation; white, z-score of 0; gray, not available). (A) The level 1 squares in this heat map (high-level functional categories) indicate the functions predicted to increase or decrease with MAGI1 depletion. The size of each box reflects the P-value (the larger the box, the smaller the P-value). The P-values for the Infectious Disease categories are within the six highest category. The z-score for the Infectious Disease category is predicted to decrease with MAGI1 depletion. (B) The hierarchical heat map of the level 1 category Infectious Disease. [file Data_Sheet_1.zip › Supplementary Figure2.tif]
